# Monitoring subthalamic oscillations for 24 hours in a freely moving Parkinson's disease patient

**DOI:** 10.1002/mds.27657

**Published:** 2019-03-20

**Authors:** Mattia Arlotti, Chiara Palmisano, Brigida Minafra, Massimiliano Todisco, Claudio Pacchetti, Andrea Canessa, Nicoló G. Pozzi, Roberto Cilia, Marco Prenassi, Sara Marceglia, Alberto Priori, Paolo Rampini, Sergio Barbieri, Domenico Servello, Jens Volkmann, Gianni Pezzoli, Ioannis U. Isaias

**Affiliations:** ^1^ Clinical Center for Neurotechnologies, Neuromodulation, and Movement Disorders Fondazione IRCCS Ca'Granda Ospedale Maggiore Policlinico Milan Italy; ^2^ Department of Neurology University Hospital and Julius Maximilian University Wuerzburg Germany; ^3^ Department of Electronics, Information and Bioengineering MBMC Lab Politecnico di Milano, Milan Italy; ^4^ Parkinson and Movement Disorder Unit National Neurological Institute Foundation “C. Mondino” IRCCS Pavia Italy; ^5^ Fondazione Europea di Ricerca Biomedica Cernusco s/N, Milan Italy; ^6^ Department of Informatics, Bioengineering, Robotics and System Engineering University of Genoa Genoa Italy; ^7^ Centro Parkinson ASST G. Pini‐CTO Milan Italy; ^8^ Dipartimento di Ingegneria e Architettura Università degli Studi di Trieste Trieste Italy; ^9^ “Aldo Ravelli” Research Center, Department of Health Sciences University of Milan and Ospedale San Paolo Milan Italy; ^10^ Department of Neurosurgery and Neurology IRCCS Galeazzi Hospital Milan Italy

Adaptive deep brain stimulation (DBS) devices aim to personalize stimulation delivery by following the current state of symptom‐specific neural signals during different activities of daily living (walking, sleeping, etc.). This approach is not yet suitable for clinical practice, and groundwork is needed. The first essential steps for establishing adaptive DBS comprise the capacity for measurements in chronically implanted patients (to avoid the “stunning effect”)^1^ and for prolonged recordings not corrupted by artifacts.^2^, ^3^

Our centers teamed up to address these challenges and were able to successfully record the bilateral subthalamic local field potentials for 24 hours in 1 patient chronically implanted for Parkinson's disease (ClinicalTrials.gov: NCT03422757). The recordings were performed in a 55‐year‐old woman suffering from akinetic‐rigid PD for 8 years and admitted to the hospital for battery replacement after 4 years of subthalamic DBS (Activa PC, lead model 3389; Medtronic). After 30‐minute recordings (baseline) in stim‐off/meds‐off condition (overnight pausing of all dopaminergic medication), we set the new AlphaDBS device (Newronika Srl)^1^ to the chronically active parameters (left: 3‐C+, 4.8 V, 60 μs, 170 Hz; right: 11‐C+, 5.5 V, 60 μs, 170 Hz). Recordings lasted for 24 hours continuously over 2 days, during which the patient freely performed everyday life activities and had approximately 6 hours of sleep at night. Recordings were performed during active stimulation in a differential configuration (left: contacts 0‐1; right: contacts 8‐9) and stored on the device.^1^ We chose these contacts as they showed the highest peak in the β‐frequency range. Despite active stimulation, we observed clear modulation of the low β‐frequency range (13‐20 Hz) following levodopa intake.^4^ In this band, we recorded the highest interhemispheric subthalamic cross‐frequency amplitude‐amplitude coupling (*r* = 0.62, *P* < 0.0001) during the daytime, which diminished during night sleep (Fig. 1). The clinical efficacy of DBS was maintained throughout the experiment, with stable improvement ranging between 30% and 37% (with respect to the baseline MDS‐UPDRS part III score of 39/108), which was similar to that experienced by the patient at home (36% improvement in stim‐on/meds‐off at enrollment visit). During the study, the patient continued the home medication regimen and took 1 pill of fast‐acting oral levodopa/benserazide 100/25 mg on 2 occasions. Levodopa improved parkinsonian symptoms by 5 points on the MDS‐UPDRS part III score, without adverse events (i.e., dyskinesias). No adverse events or complaints by the patient were reported. The Ethical Committee approved the study, and all patients gave written informed consent.

**Figure 1:**
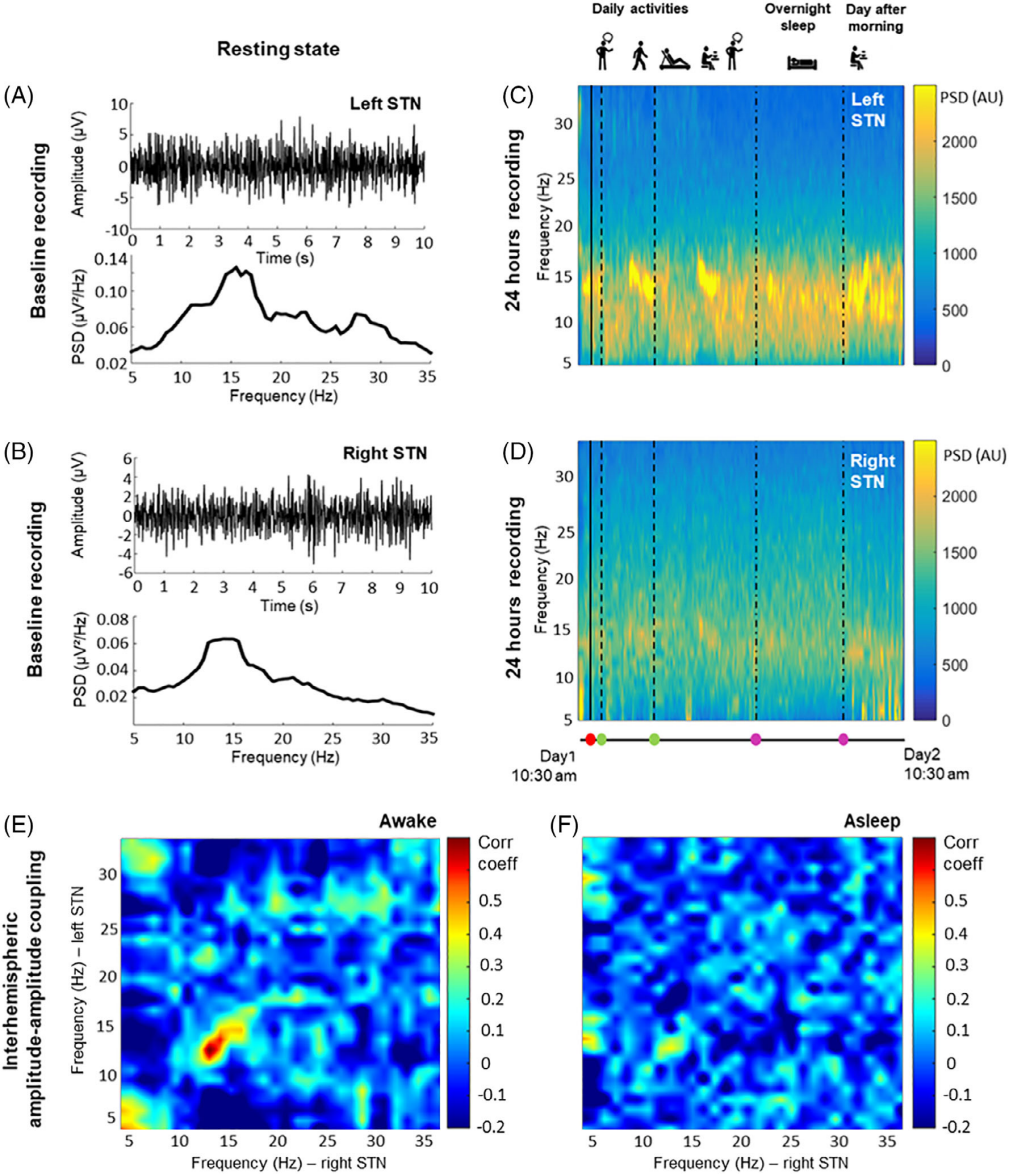
Time plot (upper row) and power spectral density (lower row) of local field potentials (LFPs) recorded in the left (A) and the right (B) subthalamic nucleus (STN) in baseline condition. Time‐frequency plot of LFPs in the range 5‐35 Hz recorded in the left (C) and the right (D) STN during 24 hours of cDBS. Red dot indicates when deep brain stimulation was activated and green dots the intake of levodopa. Nighttime sleep is shown between the 2 pink dots. Interhemispheric subthalamic cross‐frequency amplitude‐amplitude coupling during daytime (E) and nighttime sleep (F).

Our results prove the feasibility of prolonged recordings (up to 24 hours) in freely moving, chronically stimulated patients. They further corroborate the hypothesis that oscillations in the β‐frequency range might be used as a levodopa‐related biomarker for adaptive DBS paradigms, as they are present during active stimulation and years after surgery. We also provide for the first time preliminary evidence that interhemispheric subthalamic coupling changes between wakefulness and sleep can be monitored and possibly serve as an additional behavior‐specific biomarker. These findings pave the way for testing different adaptive stimulation paradigms for STN‐DBS and prompt a more accurate definition of symptom‐related and behavior‐specific biomarkers in PD.^5^

## Authors' Contributions

1) Research project: A. Conception, B. Organization, C. Execution;

2) Statistical Analysis: A. Design, B. Execution, C. Review and Critique;

3) Manuscript: A. Writing of the first draft, B. Review and Critique.

M.A.: 1B, 1C, 2A, 2B, 3A

C.P.: 1B, 1C, 2C

B.M.: 1B

M.T.: 1C

C.P.: 1B

A.C.: 1C, 2A, 2C, 3B

N.G.P.: 3B

R.C.: 1B

M.P.: 1C

S.M.: 1A, 1B, 3B

A.P.: 1A

P.R.: 1B

S.B.: 1B

D.S.: 1C

J.V.: 1A, 3B

G.P.: 1B, 3B

I.U.I.: 1A, 1B, 2C, 3A, 3B
